# Effect of losartan on performance and physiological responses to exercise at high altitude (5035 m)

**DOI:** 10.1136/bmjsem-2020-000982

**Published:** 2021-01-06

**Authors:** Samuel J E Lucas, William L Malein, Owen D Thomas, Kimberly M Ashdown, Carla A Rue, Kelsey E Joyce, Charles Newman, Patrick Cadigan, Brian Johnson, Stephen D Myers, Fiona A Myers, Alexander D Wright, John Delamere, Chris H E Imray, Arthur R Bradwell, Mark Edsell, Birmingham Medical Research Expeditionary Society

**Affiliations:** 1 School of Sport, Exercise and Rehabilitation Sciences, University of Birmingham, Birmingham, UK; 2 Department of Anaesthesia, Ninewells Hospital, Dundee, UK; 3 Department of Anaesthesia, Royal Gwent Hospital, Aneurin Bevan University Health Board, Newport, UK; 4 Occupational Performance Research Group, University of Chichester, Chichester, West Sussex, UK; 5 Royal Centre for Defence Medicine, Queen Elizabeth Hospital Birmingham, Birmingham, UK; 6 Birmingham Medical Research Expeditionary Society, Birmingham, UK; 7 School of Biological Sciences, University of Portsmouth, Portsmouth, Hampshire, UK; 8 School of Medicine, University of Birmingham, Birmingham, UK; 9 Department of Vascular Surgery, University Hospitals of Coventry and Warwickshire, Warwick Medical School, University of Warwick, Coventry, UK; 10 Department of Anaesthesia, St George's University Hospitals NHS Foundation Trust, London, UK

**Keywords:** altitude, exercise, pulmonary, cardiovascular

## Abstract

**Objective:**

Altitude-related and exercise-related elevations in blood pressure (BP) increase the likelihood of developing pulmonary hypertension and high-altitude illness during high-altitude sojourn. This study examined the antihypertensive effect and potential exercise benefit of the angiotensin II receptor antagonist losartan when taken at altitude.

**Methods:**

Twenty participants, paired for age and ACE genotype status, completed a double-blinded, randomised study, where participants took either losartan (100 mg/day) or placebo for 21 days prior to arrival at 5035 m (Whymper Hut, Mt Chimborazo, Ecuador). Participants completed a maximal exercise test on a supine cycle ergometer at sea level (4 weeks prior) and within 48 hours of arrival to 5035 m (10-day ascent). Power output, beat-to-beat BP, oxygen saturation (SpO_2_) and heart rate (HR) were recorded during exercise, with resting BP collected from daily medicals during ascent. Before and immediately following exercise at 5035 m, extravascular lung water prevalence was assessed with ultrasound (quantified via B-line count).

**Results:**

At altitude, peak power was reduced relative to sea level (p<0.01) in both groups (losartan vs placebo: down 100±29 vs 91±28 W, p=0.55), while SpO_2_ (70±6 vs 70±5%, p=0.96) and HR (146±21 vs 149±24 bpm, p=0.78) were similar between groups at peak power, as was the increase in systolic BP from rest to peak power (up 80±37 vs 69±33 mm Hg, p=0.56). Exercise increased B-line count (p<0.05), but not differently between groups (up 5±5 vs 8±10, p=0.44).

**Conclusion:**

Losartan had no observable effect on resting or exercising BP, exercise-induced symptomology of pulmonary hypertension or performance at 5035 m.

**What are the findings?**

- Losartan, an angiotensin II type-I receptor antagonist, had no observable effect on either resting or exercise blood pressure responses at 5035 m, nor did it reliably reduce exercise-induced symptomology of pulmonary hypertension or improve arterial saturation.
- Losartan did not improve exercise performance at 5035 m.

**How might it impact on clinical practice in the future?**

- Findings of a null effect on blood pressure management has implications for hypertensive individuals currently receiving antihypertensive treatment who travel to high altitudes, as they may be at greater risk for high altitude illness and cardiovascular events than the normotensive individuals tested herein.

## Introduction

Altitude and dynamic exercise both increase arterial blood pressure (BP).^1 2^ This information is of increasing relevance to standard clinical practice given the increasing numbers of individuals with pre-existing cardiovascular conditions (eg, hypertension) sojourning at high altitude.^3^ Moreover, BP management is essential in the high-altitude setting since pulmonary hypertension can precipitate the development of high altitude pulmonary oedema (HAPE).^4^ The worrying symptomology of HAPE, including fatigue, dyspnoea and the potential for death, has made prophylaxis and treatment an important aspect of modern altitude medicine.^5^ The antihypertensive actions of nifedipine and dexamethasone have been shown to decrease arterial BP (although not consistently^6^), pulmonary artery pressure, and reduce the incidence of HAPE.^6 7^

Pharmacological inhibition of the renin-angiotensin-aldosterone system (RAAS) could also be beneficial for HAPE prevention and may also improve exercise performance at altitude. Support for how RAAS inhibition may prevent HAPE lies within genetic studies, which have demonstrated that genetic polymorphisms among RAAS-associated candidate genes are related with the physiologic response to hypoxic exposure.^8 9^ For example, polymorphisms in the ACE gene (eg, I/I vs D/D genotype) appear to partly contribute to the heterogeneity displayed in the susceptibility to high-altitude illness.^10^ Further, the I allele is more frequently found in climbers who successfully reach over 8000 m and has been associated with the ability to maintain higher arterial oxygenation at altitude, a greater hypoxic ventilatory drive, higher endurance performance at sea level, as well as better high-altitude adaptation.^8 10 11^ Parati *et al* have previously demonstrated the anti-hypertensive effects of RAAS inhibition (via telmisartan, 80 mg dose) during exposure to high altitude,^12^ however, the potential of RAAS inhibition to improve exercise performance at altitude remains unknown, as does its ability to reduce exercise-induced evidence of pulmonary hypertension (eg, increases in extravascular lung water (EVLW)).^13^ Interestingly, Kiely *et al* reported that losartan, an angiotensin II receptor antagonist, reduced both systemic BP and pulmonary vascular resistance in response to acute graded hypoxic exposure.^14^ Whether this laboratory-based observation translates to the sustained hypoxic exposure of the high-altitude setting remains to be determined.

Therefore, the purpose of this study was to evaluate the effects of the angiotensin II receptor antagonist, losartan, on BP during ascent and to determine whether it could improve exercise performance at 5035 m. We hypothesised that blockade of the angiotensin receptor would reduce altitude-related increases in resting and exercising BP, reduce the effect of strenuous exercise on the development of EVLW at high altitude, and ultimately improve exercise performance via enhanced pulmonary ventilation (V_E_)-perfusion matching and increased arterial oxygenation.

## Materials and methods

### Design and participants

Twenty participants (14 male, 6 female) free of any pre-existing conditions were included in the study. None of the participants had slept at high altitude (ie, >2500 m) in the 3 months prior to the experiment. ACE genotyping (II, ID or DD) was performed for each participant prior to pair-matching participants for ACE genotype, age, sex and previous altitude exposure. Following matching, a double-blind, randomised, placebo-controlled trial design was adopted in which individuals within each pair were randomly assigned to either placebo or losartan groups. Losartan (100 mg/day or placebo) and placebo (starch) administration began in the UK 21 days prior to departure for Quito, Ecuador (2850 m). On arrival at Quito, participants ascended over 8 days to the Whymper Hut on the flank of Chimborazo volcano (5035 m). Within 48 hours of arrival to 5035 m, each pair of participants (within ~1 hour of each other) completed a graded maximal aerobic capacity exercise test. Further details of participant genotype pairing, losartan administration and ascent profile have been previously published as part of another study conducted on this expedition.^15^

### Baseline and daily measures

Baseline measures of BP and exercise performance were recorded at sea-level and before losartan/placebo administration (~4 weeks prior to ascent). During ascent, measures of resting systolic and diastolic BPs (SBP and DBP) were collected each morning using a manual sphygmomanometer as part of the routine medical examination.

### Exercise protocol and measures

#### Sea-level exercise test

Baseline sea-level maximal exercise tests were conducted 4 weeks prior to ascent. This test consisted of a graded exercise test to volitional fatigue^16^ on a cycle ergometer (Alticycle) designed for altitude studies.^17^ Maximal power output (Watt_max_) was determined and heart rate (HR) recorded via short range telemetry (Polar Electro, UK). These data were used to calculate the intensity steps for high-altitude exercise tests and to determine maximal power output for this exercise modality (supine cycling).

#### Altitude exercise test

Participants were instrumented and then rested for at least 2 min while pre-exercise measurements of oxygen uptake (VO_2_), carbon dioxide production, V_E_, HR, SBP, and DBP were collected. Participants undertook a 5 min self-paced warm-up and a modified graded exercise test on the Alticycle. This modified graded exercise test began at an intensity of 30% of sea-level Watt_max_, with 10% increases every 3 min up to 80% of sea-level Watt_max_, followed by 10% increases every minute until volitional fatigue. Expired respiratory gases were analysed breath-by-breath using a Cosmed K4b^2^ (Metabolic Company, Rome, Italy) portable metabolic system alongside continuous measurements of HR (via three-lead ECG), pulse oximetry (oxygen saturation (SpO_2_), Datex Ohmeda 3900, GE Healthcare, USA), and beat-to-beat measurements of SBP and DBP by photoplethysmography (Portapres, Finapres Medical Systems BV, Netherlands). The exercise-induced change in SBP and DBP is reported, calculated as the difference between pre-exercise and value obtained at Watt_max_.

### EVLW assessment

To assess EVLW, lung ultrasound was used to detect the presence of ultrasound B-lines,^18^ with these assessments performed at 5035 m, immediately prior to and immediately after exercise tests. Ultrasounds were conducted using a portable system (MicroMaxx, Sonosite, Bothell, Washington, USA) in conjunction with an 8 MHz linear transducer. These examinations consisted of an 8-zone technique, scanning in the mid-clavicular line in the second and third intercostal spaces and the mid-axillary line in the fifth and sixth intercostal spaces each side.^18^ These points of insonation were marked with permanent ink prior to testing to minimise time and ensure the same zones were scanned on each examination. Ultrasound examinations were performed by two clinicians experienced with this methodology, and pre-exercise and postexercise scans for each participant were done by the same operator. Ultrasounds were recorded live (Nikon D3S, Japan, on video mode) without audio comments. Recordings only included participant number and time-point of examination (ie, first or second ultrasound). No indications for the presence or number of B-Lines were made at the time of the ultrasound nor at any time during the expedition. Rather, recordings were analysed on return to the UK by two blinded experimenters at separate sites. The total number of B-lines from each scan was taken as the average from these two reviewers. The inter-reviewer correlation on B-line count was strong (R=0.92). Group allocation was only revealed once recordings had been analysed and all B-lines counted.

### Data analysis

Independent t-tests were used to test for differences between groups at rest and exercise-related time points of interest at 5035 m (when measures were not repeated), such as, at peak power, immediately postexercise or exercise-induced change. Repeated-measures analysis of variance with pairwise comparisons (Bonferroni corrected) was used for between-group comparisons of the morning BP assessment during ascent. All data are presented as mean±SD and statistical significance was set at p<0.05.

### Patient and public involvement

This study was supported by the Birmingham Medical Research Expeditionary Society, which provided input for the conduct of the research. Patients were not included. Public involvement was limited to recruitment. Notification was given to participants at the time of consent that acquisition of personal data was permitted on request. Permission was also obtained at this time for the dissemination of deidentified data within the research team and only externally when a reasonable request was submitted directly to the corresponding author of the present study within 6 months of its publication. A portion of the cohort was invited to review the research methods for accuracy and readability.

Participants give their written informed consent and the study was conducted in agreement with Declaration of Helsinki principles. This study did not aim to investigate safety or efficacy of the already Food and Drug Administration (FDA)-approved drug included, thus no clinical trial approval was obtained. There were no active FDA recalls for the drug for the duration of the study.

## Results

Two paired participants did not complete the exercise test at high altitude, withdrawn during submaximal intensity steps due to symptomatic abnormalities in the ECG recording (unifocal ventricular ectopics and atrial tachycardia) and very low SpO_2_ measures (ie, <55%). For these two participants, post-exercise ultrasound scans were still conducted following test termination, so EVLW data were collected from all 20 participants. For the remaining exercise-related measures, data are reported from 18 paired participants, unless otherwise stated. Such cases related to participant’s exercising BP recordings being deemed unreliable (eg, Portapres BP signal dropped out, n=2) and a missing ultrasound data file (n=1), whereby group comparisons were made without the matched pair for this measure.

### Ascent and resting measures at 5035 m

**Figure 1:**
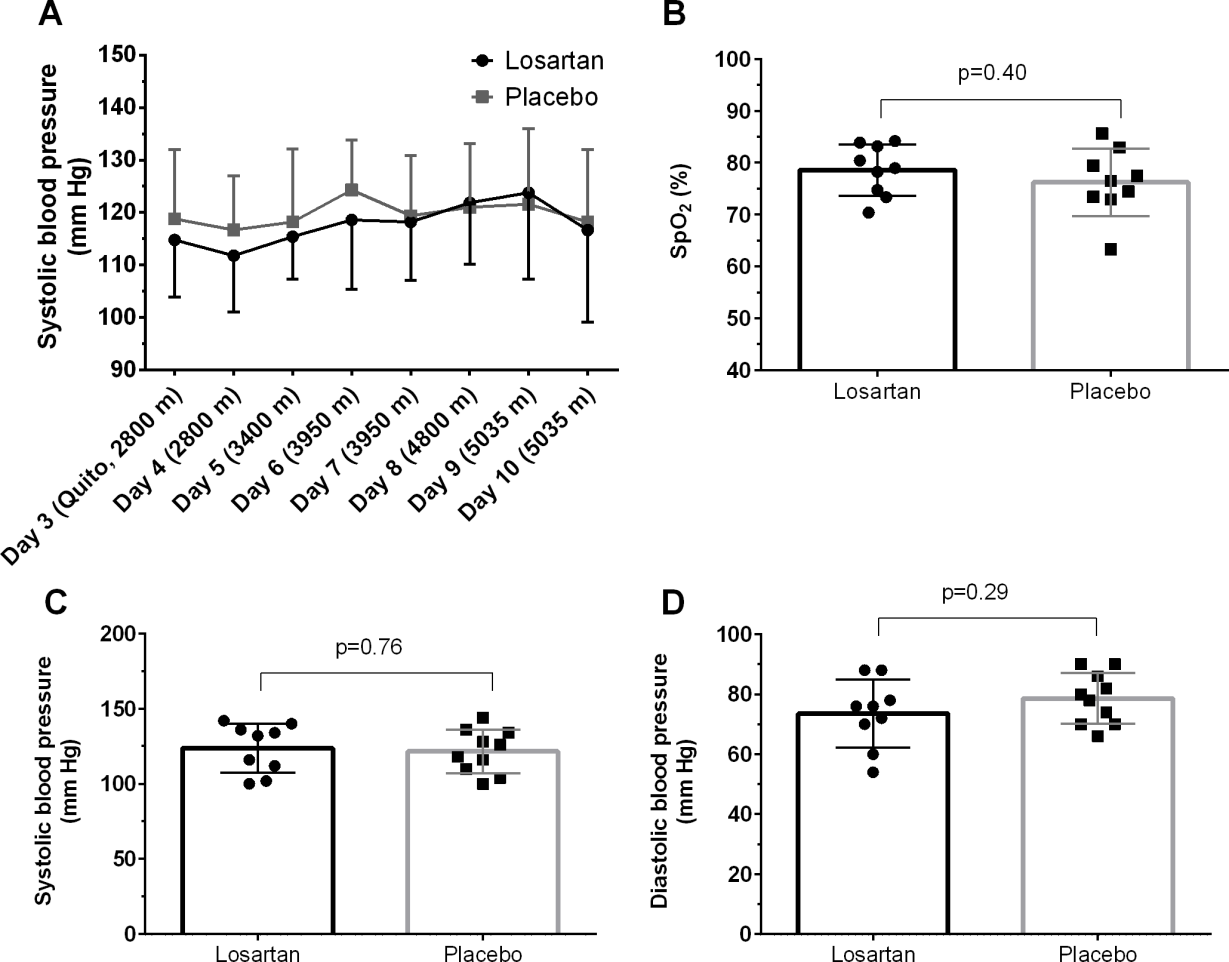
Measures (mean±SD) of resting systolic blood pressure during ascent and arrival to 5035 m (A, n=10 matched pairs), and measures of peripheral arterial oxygen saturation (SpO_2_, (B), systolic (C) and diastolic (D) blood pressure measures from the first morning routine medical examinations at 5035 m.

During ascent, there was no significant difference between groups for morning resting measures of SBP (group effect: p=0.71), although SBP was on average lower in the losartan group at the lower altitudes (figure 1A). On average, SBP increased during ascent, although not significantly so (p=0.17).

At 5035 m, there was no significant difference between groups for resting measures of SpO_2_ (losartan vs placebo: 79±5 vs 76±6%, p=0.40), HR (80±12 vs 76±15 b/min, p=0.50), or SBP (124±16 vs 122±14 mm Hg, p=0.76; figure 1).

### Exercise at 5035 m: performance and exercise-induced responses

**Figure 2:**
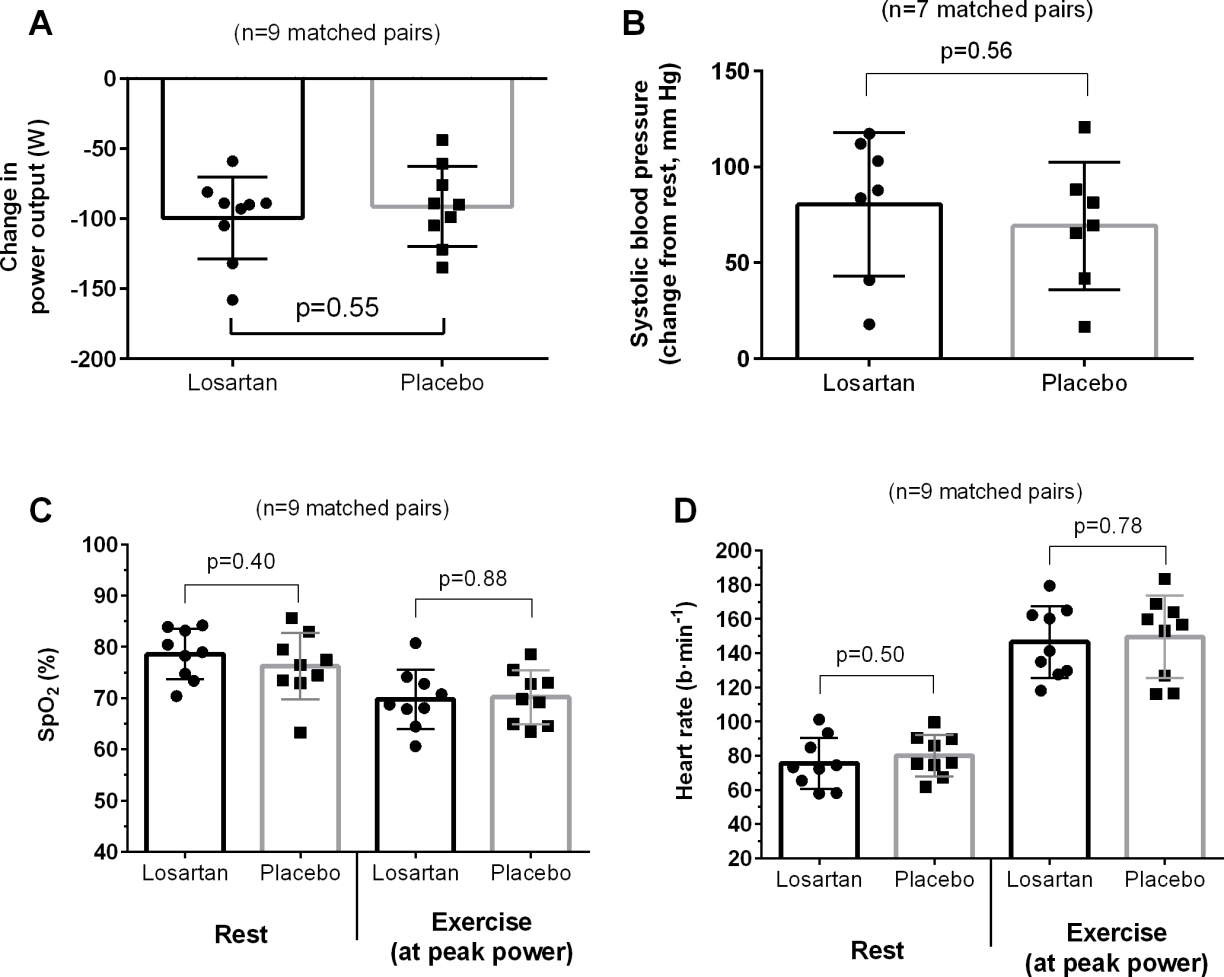
Comparison of between losartan and placebo groups for the reduction in peak power at 5035 m relative to individuals sea-level performance (A, n=9 matched pairs), the exercise-induced increase in systolic blood pressure (B, n=7 matched pairs), peripheral arterial oxygen saturation (SpO_2_, (C) and heart rate (D) at rest and at peak power (n=9). Data are presented as mean±SD. Lower sample size for blood pressure measures were due to unreliable data produced from the finometer in at least one matched pair.

Peak power was reduced at high altitude relative to sea-level values (p<0.01), with this reduction in power similar for both losartan and placebo groups (down 100±29 vs 91±28 W, respectively, p=0.55; figure 2). At peak power, measures of SpO_2_ (70±6 vs 70±5%, p=0.88), VO_2peak_ (31.3±4.6 vs 34.0±7.2 mL/kg/min, p=0.35), V_E_ (142±38 vs 146±31 L/min, p=0.81) and HR (146±21 vs 149±24 b/min, p=0.78) were similar between groups. The increase in SBP from rest to peak power was also no different between groups (n=14 paired participants: increased by 80±37 vs 69±33 mm Hg, p=0.56).

**Figure 3:**
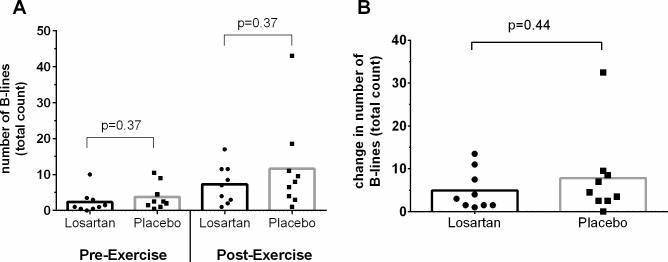
Prevalence of extravascular lung water, as indexed by number of ultrasound B-lines, in response to a graded exercise test to volitional fatigue at 5035 m. (A) shows absolute count at rest before and then immediately following exercise. (B) shows change in number of B-lines induced by the exercise test.

Ultrasound B-lines were present in 18/19 participants (one pre-exercise scan was lost) prior to the exercise test at 5035 m, and present in all 20 participants immediately following exercise (figure 3A). Exercise increased B-line count in both groups, however, there was no significant difference between groups at either the pre-exercise or postexercise time points, nor for the change in B-lines from resting values (up by: 4.9±4.6 (losartan) vs 7.8±9.8 (placebo), p=0.44; figure 3B).

## Discussion

Exposure to high altitude induces hypertension, both systemic and pulmonary, which exacerbates arterial hypoxia by means of increasing pulmonary vasoconstriction and V_E_ perfusion mismatching.^1^ Given that exercise also increases BP, especially at higher intensities,^19^ the combination of these two stimuli may exacerbate the likelihood of pulmonary hypertension developing into high altitude illness (ie, HAPE), but may also be a mechanism by which exercise capacity is reduced at high altitude. Therefore, we sought to determine the anti-hypertensive effectiveness of losartan to lower altitude-induced systemic and pulmonary hypertension and improve exercise performance at high altitude (5035 m). Our findings, however, showed no beneficial effect of RAAS inhibition via this angiotensin II antagonist on resting and exercising BP responses, SpO_2_, exercise-induced symptomology of pulmonary hypertension, or indeed exercise performance at 5035 m. These findings reflect the complex nature of this physiological response and highlight the need for further research to understand the mechanisms and consequences of hypoxic pulmonary vasoconstriction on high-altitude performance.^20^

The lack of effect on resting BP we observed is consistent with findings from Parati and colleagues.^12^ Specifically, while they did show a lowering of ambulatory BP at sea level and at 3400 m with the use of the angiotensin-II receptor antagonist, telmisartan, its antihypertensive efficacy was not evident at 5400 m, similar to our current observations at 5035 m. Interestingly, although our group differences did not reach statistical significance, the losartan group also had lower SBP at altitudes below 3950 m. Taken together, both studies indicate that these anti-hypertensive medications are not effective at higher altitudes (eg, >3500 m). These findings have important potential clinical implications for hypertensive individuals who wish to travel to high altitudes (eg, >4000 m) that are currently receiving antihypertensive treatment, the numbers of which are increasing.^3^ Therefore, a pharmacological strategy to appropriately treat and manage the combined hypertensive effects of hypoxia and exercise is warranted and deserves further study.

Our findings illustrate the utility of using lung ultrasound to assess EVLW.^13 21^ The presence of B-lines observed at rest, although low, supports previous observations that indicate a degree of interstitial oedema is frequent at such altitudes in both healthy individuals and those with mountain sickness.^18 21^ The almost universal (19/20) increase in EVLW following strenuous exercise further implicates exercise as a risk factor for developing HAPE and may represent what is increasingly being considered as subclinical HAPE.^18^ Eldridge *et al* demonstrated bronchoalveolar lavage samples from healthy individuals after heavy exercise in hypoxia that mirrored the composition of that seen in early HAPE, which persisted for at least 26 hours postexercise.^22^ Further, our group have previously demonstrated that ultrasound evidence of exercise-induced pulmonary oedema at 4000 m persisted for approximately 4 hours following exercise before returning to baseline.^13^ Taken together, these findings indicate an additional pathological process is required for the propagation and persistence of fulminant HAPE on top of a propensity to develop oedema following exercise.

Justification for our choice of the angiotensin-II receptor antagonist, losartan, over other agents that influence aspects of the RAAS, was based on previous findings that highlighted angiotensin-II’s specific ability to modulate acute pulmonary vasoconstriction responses.^14 23^ Furthermore, genetic studies have revealed the involvement of several genes associated with the RAAS in relation to the physiological response to hypoxia, which have included the AGRT1 gene.^8 24^ Polymorphisms within the AGRT1 have specifically been associated with the risk for developing HAPE.^10 25 26^ Thus, we hypothesised that the administration of losartan would elicit favourable responses in systemic and pulmonary pressures at high altitude and improve exercise capacity. Ultimately, we aimed to determine if losartan could be a beneficial agent for the prophylactic treatment of HAPE and offset altitude-related impairments in exercise capacity. However, based on the findings of the current study, a daily dose of losartan at 100 mg appears not to be useful in this context. Finally, the development of pulmonary oedema at altitude is likely polygenetic and multifactorial, thus blockage of a single pathway may not be sufficient. It may be that a specific pulmonary vasodilator (eg, nifedipine, a calcium channel blocker) or a type-5 phosphodiesterase inhibitor (eg, tadalafil), which have been shown to reduce incidence of HAPE among susceptible individuals,^6 7^ is required to prevent exercise-induced pulmonary hypertension and improve exercise performance at altitude.

### Limitations

The relatively small sample size limited this study’s power to detect a significant effect of losartan on resting BP and prevalence of B-lines induced by exercise. Parati *et al*’s study demonstrated the lower BP effect at 3400 m in a sample of 20 taking telmisartan (vs 25 in placebo group). Our study sample size was restricted due to difficulties and cost of recruiting participants to a remote place for an extended time, typical of this type of field research.^27^ Second, we did not perform lung ultrasound at sea level to assess effect of strenuous sea-level exercise on the development of B-lines. However, we have previously reported that EVLW is not detected at sea level before or after exercise to exhaustion in a similar group of individuals as studied here.^13^ The differential diagnosis for B-lines includes pneumonitis and interstitial pneumonia. However, the pattern of B-lines was typical of pulmonary oedema, and our participants were otherwise well with no history of chronic lung disease and the observed increase is most likely explained by worsening interstitial oedema.

## Conclusion

Our findings showed that the angiotensin II type-I receptor antagonist, losartan, had no observable effect on either resting or exercise BP responses at 5035 m, nor did it reliably reduce exercise-induced symptomology of pulmonary hypertension (as assessed via ultrasound quantification of EVLW) or improve arterial saturation. These null effects were supported by no observable difference in exercise performance. Our findings have potential clinical implications for hypertensive individuals who travel to high altitudes (eg, >4000 m) that are currently receiving antihypertensive treatment, as they may be at greater risk for high altitude illness and cardiovascular events since their anti-hypertensive treatments appear to exhibit a null effect on BP management.

## Data Availability

Data are available on reasonable request. The data are owned by the University of Birmingham and can be obtained on reasonable request from either the corresponding author or the School of Sport, Exercise, and Rehabilitation Sciences +44 (0)121 414 9286. Reuse is not permitted unless otherwise indicated at the time of reasonable request. There is no additional relevant information.
